# Development of Antioxidant Protein Extracts from Gilthead Sea Bream (*Sparus aurata*) Side Streams Assisted by Pressurized Liquid Extraction (PLE)

**DOI:** 10.3390/md19040199

**Published:** 2021-04-01

**Authors:** Beatriz de la Fuente, Noelia Pallarés, Houda Berrada, Francisco J. Barba

**Affiliations:** Preventive Medicine and Public Health, Food Science, Toxicology and Forensic Medicine Department, Faculty of Pharmacy, Universitat de València, Avda. Vicent Andrés Estellés, 46100 Burjassot, València, Spain; beatriz.fuente@uv.es (B.d.l.F.); noelia.pallares@uv.es (N.P.)

**Keywords:** pressurized liquid extraction, gilthead sea bream, side streams, protein, SDS-PAGE, antioxidant capacity, mycotoxins, heavy metals

## Abstract

The pressurized liquid extraction (PLE) technique was used, for the first time, to obtain protein extracts with antioxidant activity from side streams (muscle, heads, viscera, skin, and tailfins) of gilthead sea bream (*Sparus aurata*) in order to give added value to these underutilized matrices. Extraction conditions previously optimized for sea bass (*Dicentrarchus labrax*) side streams were applied. Protein recovery percentages were 22% (muscle), 33% (heads), 78% (viscera), 24% (skin), and 26% (tailfins), which represented an increase of 1.2–4.5-fold compared to control samples (extraction by stirring). The SDS-PAGE profiles revealed that PLE-assisted extraction influenced protein molecular weight distribution of the obtained extracts. PLE conditions also allowed increasing the antioxidant capacity measured by both Trolox equivalent antioxidant capacity (TEAC; 1.3–2.4 fold) and oxygen radical absorbance capacity (ORAC; 1.9–6.4) assays for all fish extracts. Inductively coupled plasma mass spectrometry (ICP-MS) and high-performance liquid chromatography coupled with electrospray ionization quadrupole time-of-flight mass spectrometry (LC-ESI-qTOF-MS) were used to investigate the presence of toxic metals and mycotoxins in sea bream side streams. The levels of As, Hg, Cd, and Pb were below those established by authorities for fish muscle for human consumption (except for Cd in viscera samples). Through a nontargeted screening approach, no mycotoxins or related metabolites were detected for all sea bream side streams. This study contributes to the research on the valorization of fish processing side streams using environmentally friendly technology.

## 1. Introduction

The European Union (EU) is the world’s second largest trader of fishery and aquaculture products after China [1]. The increasing importance of the European aquaculture sector is due to the increased production of high-value species. For instance, the production of the main commercial species such as salmon, bluefin tuna, sea bass, and sea bream has increased by 11% over the last decade. In terms of value, one of the most significant growths in recent years was obtained for European gilthead sea bream (*Sparus aurata*), reaching 94.936 tons and 485 million EUR in 2017 [2]. This trend is supported by the increased demand, which, together with the consumer growing interest in convenience products, has led to a greater manufacture of gutted and filleted sea bream [3]. In this sense, Pateiro et al. [4] reported that discards accounted for ~60% of the whole sea bream after the filleting process. Therefore, an increase in gilthead sea bream side streams in the upcoming years is expected. 

The relevance of fish processing side streams as an alternative source of nutrients and bioactive compounds for the food and feed industries is increasing the research on nutritional characterization and the presence of possible contaminants in these underutilized raw materials [5,6]. In this context, the nutritional composition of several sea bream side streams was recently evaluated [4,5]. The authors concluded that sea bream side streams had a significant protein, fat, and mineral content. The nutritional profiles also showed a percentage of essential amino acids close to 50%, as well as a higher content of mono- and polyunsaturated fatty acids compared to saturated ones. Therefore, they suggested sea bream side stream materials as a promising source of valuable compounds to be exploited for human consumption. In this way, fish protein hydrolysates with a remarkable essential amino-acid profile were produced from sea bream filleting side streams and were considered as a suitable tool for developing food additives and supplements [3]. Currently, only industrially processed feeds are used to grow gilthead sea bream in aquaculture systems [7]. Ingredients such as corn, wheat, pea, and soybean represent protein sources for farmed sea bream feeding [7,8]. Therefore, evaluating the transfer of feed-borne mycotoxins to different sea bream tissues is recommended. In addition, trace metals may also be transferred from the aquaculture environment to farmed fishes [6,9]. Toxic metals such as As, Hg, Cd, and Pb and mycotoxins have been screened in several side streams of sea bream, sea bass, meager, and salmon, although levels found of these contaminants were below the limits stablished by authorities [6,9,10,11]. 

The Horizon 2020 program stands for research and innovation to reach Europe’s global competitiveness, encouraging the introduction of sustainable technologies to fisheries and aquaculture [12]. One of the Horizon 2020 challenges is the recovery of proteins from natural underexploited resources in a sustainable way. Fish processing side streams have been considered ideal candidates for protein recovery due to their relevant content protein, wide availability, and low cost [13,14]. Among the different techniques developed to recover and produce proteins from marine organisms and related byproducts, solvent and enzymatic extraction processes are preferred for production of proteins of high nutritional quality with bioactive and functional properties [14]. The application of pressurized liquid extraction (PLE) to obtain protein extracts with antioxidant activity from sea bass processing side streams was recently investigated [10]. The use of water as sustainable solvent and the optimization of extraction conditions resulted in a protein recovery of 18% to 61%, depending on the fish raw material. The optimal fish extracts obtained also showed in vitro antioxidant capacity. 

It should be noted that PLE is currently considered a fast and easy extraction process, as well as an important technology for recovering a great variety of compounds from different food matrices [15,16]. PLE is based on the use of high pressure and temperature to improve the extraction efficiency by increasing the diffusion rate and solubility of analytes [16,17,18]. However, to achieve this, the optimization of different additional parameters is also required. For instance, dispersing agents are used to reduce particle clumping and solvent channeling in the extraction cell [17]. As the number of extraction cycles and the total extraction time are related to the contact time between matrix and solvent, they should be carefully selected [16]. In addition to the physicochemical properties, the nontoxicity of the solvent is a crucial factor for the sustainable recovery of food compounds, which is why the use of water as a solvent is recommended. 

Since sea bass and sea bream are considered to be closely related species, similar results would be expected. Therefore, the main objective of the present study was to apply, for the first time, the PLE technique to obtain antioxidant protein extracts from sea bream processing side streams in a sustainable way. Muscle left over, heads, viscera, skin, and tailfins of gilthead sea bream were selected as a valorization approach for these underutilized raw materials. Protein recovery, protein molecular weight distribution, and antioxidant capacity were evaluated in PLE extracts. In order to provide additional data on potential contaminants, the determination of As, Hg, Cd, and Pb, as well as a multi-mycotoxin screening in sea bream side streams, was also carried out. Overall, this study contributes to the research on the valorization of fish processing side streams using environmentally friendly technology.

## 2. Results and Discussion

### 2.1. Protein Recovery Percentage

**Figure 1:**
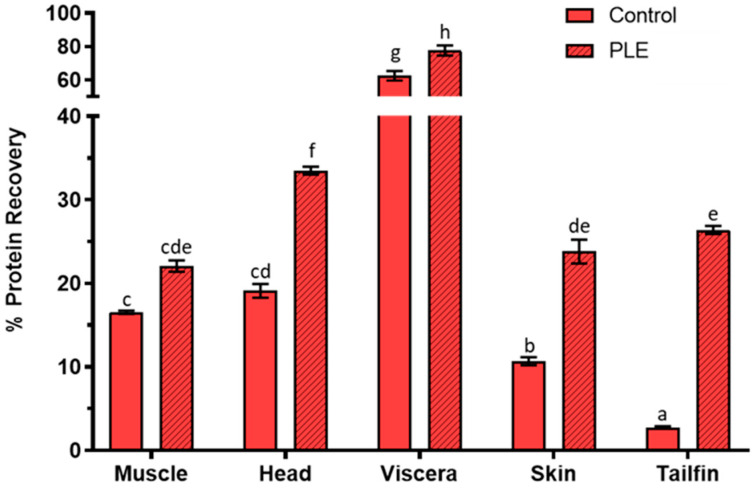
Percentage protein recovery in control and pressurized liquid extraction (PLE) extracts from muscle, head, viscera, skin, and tailfin of gilthead sea bream. Results are expressed as the mean ± standard deviation (*n* = 2). Different lowercase letters above the bars indicate statistically significant differences (*p* < 0.05) among samples.

The results of protein recovery in control and PLE extracts from side streams of gilthead sea bream are shown in Figure 1. The percentage protein recovery in PLE extracts of sea bream muscle, head, viscera, skin, and tailfins was 22.06 ± 0.68, 33.48 ± 0.47, 77.66 ± 3.01, 23.80 ± 1.43, and 26.37 ± 0.48, respectively, while it was 16.52 ± 0.20, 19.09 ± 0.84, 62.46 ± 2.85, 10.67 ± 0.49, and 2.73 ± 0.15 in the corresponding control extracts. Therefore, PLE-assisted extraction improved the protein recovery (*p* < 0.05) for all side streams, except for muscle remains. In addition, the protein yield of fish extracts increased from 1.2-fold (viscera) to 9.6-fold (tailfin). The best recovery was observed in viscera, in agreement with the protein recoveries previously obtained by PLE from sea bass side streams [10]. Similar protein recovery was observed for muscle and tailfins for both species, while higher percentages of protein recovery were found in sea bream head, viscera, and skin compared to sea bass. The PLE technique was also used to extract proteins from freeze-dried seaweeds and pepper seeds, showing protein recovery percentages of <5% and 52%, respectively [19,20]. 

### 2.2. Protein Molecular Weight Distribution

**Figure 2:**
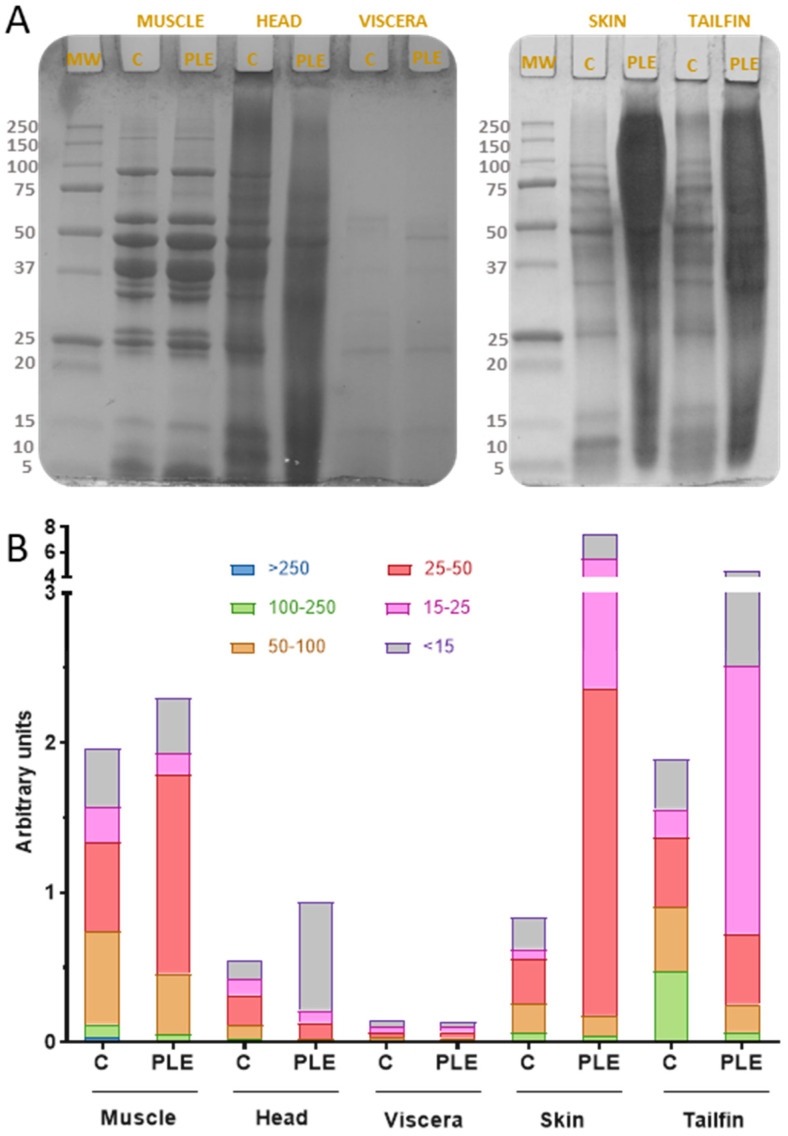
Protein molecular weight distribution of control and PLE extracts from sea bream side streams. SDS-PAGE protein profiles (**A**) and molecular weight ranges for band areas (**B**). MW: molecular weight standard. C: control extract. PLE: extract obtained by pressurized liquid extraction.

SDS-PAGE provided the electrophoretic pattern of side stream extracts of gilthead sea bream obtained by both conventional stirring and PLE-assisted extraction (Figure 2A). The gel image was evaluated through the ImageJ Program not only to obtain the molecular weight of each band but also to group the areas of the bands by kDa ranges (Figure 2B). On the one hand, different protein profiles can be observed according to the matrix studied, probably due to the particular protein composition of each side stream. On the other hand, some differences between the bands of PLE extracts with respect to the bands of control extracts were found for all sea bream side streams. Both results are in agreement with those recently reported by de la Fuente et al. [10] for protein extracts of sea bass side streams obtained by PLE.

Several clear bands (8–166 kDa) were observed for both control and PLE muscle extract profiles. This similarity could be due to the fact that both extractions were carried out at room temperature. However, the differences in the width of the bands revealed that PLE-assisted extraction provided more amount of total protein fragments, mainly in the ranges 25–50 kDa and below 15 kDa. In general, the electrophoretic patterns of sea bream muscle extracts are in agreement with those of sea bass, catfish, carp, and mackerel protein muscle [10,21,22]. The head proteins (PLE) exhibited bands from 8 to 103 kDa while those of head control extract were from 8 to 86 kDa. This range of molecular weights was equivalent to that obtained in sea bass head PLE extracts (10–85 kDa), as well as similar to parrotfish head hydrolysates subjected to hydrolysis at pH 9 for 24 h (17–76 kDa) [23]. Few differences in protein profiles of sea bream viscera extracts obtained by PLE and conventional stirring were observed. The protein bands higher than 60 kDa in the control extract were not found in the PLE extract. 

In addition, protein fragments in sea bream viscera extracts (PLE) ranging from 8 to 61 kDa were also found in sea bass viscera extracts (PLE) and unhydrolyzed cod viscera proteins [10,24]. Regarding skin, there were several bands (10–108 kDa) in control extracts, whereas few (10–62 kDa) were in PLE extracts. These protein molecular weight ranges, especially for control extracts, are in agreement with those found in PLE extracts from sea bass skin [10].

According to the gel image study, extraction of proteins from sea bream skin by PLE resulted in more protein fragments of molecular weight from 15 to 50 kDa compared to conventional extraction. The tailfin extracts showed the same behavior, which might be due to the main tissue components and the temperatures applied (55 °C for skin and 60 °C for tailfin). More protein fragments were obtained in PLE extracts compared to controls, highlighting the increase in protein recovery reached by PLE. The SDS-PAGE profiles revealed that PLE-assisted extraction influenced the protein molecular weight distribution of the obtained extracts.

### 2.3. Total Antioxidant Capacity

**Figure 3:**
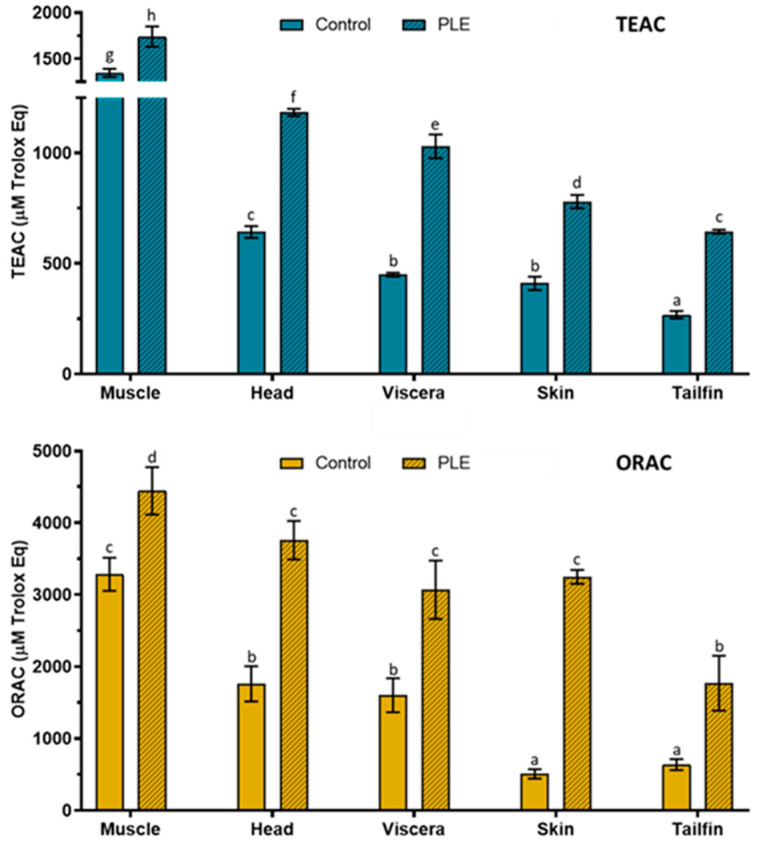
Total antioxidant capacity according to Trolox equivalent antioxidant capacity (TEAC) and oxygen radical absorbance capacity (ORAC) assays in control and PLE extracts from muscle, head, viscera, skin, and tailfin of gilthead sea bream. Results are expressed as the mean ± standard deviation (*n* = 3 for TEAC and *n* = 6 for ORAC). Different lowercase letters above the bars indicate statistically significant differences (*p* < 0.05) among samples.

The results of total antioxidant capacity determined by Trolox equivalent antioxidant capacity (TEAC) and oxygen radical absorbance capacity (ORAC) assays in control and PLE extracts of sea bream side stream extracts are shown in Figure 3. Total antioxidant capacity (TEAC and ORAC) was higher in PLE extracts than control extracts for all samples. 

TEAC values in PLE extracts were 1739 ± 111, 1184 ± 16, 1030 ± 54, 780 ± 30, and 644 ± 9 µM Trolox equivalent (Eq) for muscle, head, viscera, skin, and tailfins, respectively, while values in the corresponding control extracts were 1345 ± 44, 649 ± 27, 449 ± 9, 410 ± 30, and 268 ± 17 µM Trolox Eq. Similarly, ORAC values (µM Trolox Eq) in PLE extracts were 4445 ± 331 (muscle), 3758 ± 269 (heads), 3069 ± 407 (viscera), 3247 ± 97 (skin), and 1769 ± 382 (tailfins), whereas control ORAC values were 3284 ± 230, 1761 ± 246, 1601 ± 236, 506 ± 66, and 637 ± 77, respectively. It is known that a shorter protein chain results in a greater antioxidant capacity. Therefore, the antioxidant capacity of the sea bream extracts could be related to the protein profile previously analyzed by SDS-PAGE, since the PLE extracts contained more protein fragments of lower molecular weight than the control extracts. The highest antiradical activity was found in muscle followed by head, viscera, skin, and tailfin extracts for both antioxidant methods. According to these results, PLE conditions allowed increasing the antioxidant capacity according to both TEAC (1.3–2.4 fold) and ORAC (1.9–6.4) of sea bream PLE extracts compared to controls. In general, a similar behavior of antiradical activities was obtained from TEAC and ORAC in sea bass side streams extracts [10]. For instance, the highest values were found in muscle, while the lowest were found in tailfins for both fish species. It should be noted that the values obtained (TEAC and ORAC) in PLE extracts of gilthead sea bream were higher than those reported in PLE extracts of sea bass for side streams despite the application of the same extraction conditions. In addition to protein chain size, the presence of some polar amino acids could also contribute to antioxidant capacity [25]. In this sense, tyrosine, histidine, and lysine were recently determined in the muscle, gills, viscera, and skin of gilthead sea bream [4].

Therefore, this study revealed a matrix effect in PLE-assisted extraction as greater protein recovery and antioxidant capacity were obtained for sea bream side streams compared to sea bass. However, for SDS-PAGE profiles, the electrophoretic pattern followed was quite similar.

### 2.4. Determination of Heavy Metals and Mycotoxins in Side Streams of Gilthead Sea Bream

Heavy-metal concentrations, including As, Hg, Cd, and Pb in muscle, head, viscera, skin, and tailfin of gilthead sea bream are shown in Table 1. Mean concentration ranges, expressed as µg/g of wet weight (ww), were 0.4406–2.5865, 0.0422–0.0886, 0.0008–0.0683, and 0.0054–0.0614 for As, Hg, Cd, and Pb, respectively. For muscle and head samples, the most abundant element was As followed by Hg, Pb, and Cd. As for skin and tailfin side streams, the most abundant element was also As, followed by Pb, Hg, and Cd. Regarding viscera, the decreasing order of toxic metals was As>Cd>Hg>Pb. These results are comparable to those previously published about side streams of farmed gilthead sea bream. For instance, Kalantzi et al. [9] reported concentration ranges for As, Hg, Cd, and Pb in muscle, gills, liver, and intestines. Arsenic content was higher in muscle (0.98–2.99 µg/g) and gills (0.93–1.21 µg/g), while it was equivalent in viscera (1.77–2.64 µg/g). The authors found lower Hg levels in gills (0.001 µg/g), as well as similar levels in muscle (0.02–0.10 µg/g) and viscera (0.03–0.05 µg/g). With regard to Cd, the values ranged from 0.12 to 0.26 µg/g for the viscera sample, being higher than those shown in this study. As for Pb concentration, the data for both gills (0.03–0.04 µg/g) and viscera (0.05 µg/g) were also higher. In the same way, the boxplots of toxic metals of muscle, head, gills, viscera, and skin described by Kandyliari et al. [6] showed higher concentrations of As, Cd, and Pb in muscle tissue. They also observed higher levels of As in skin, as well as Cd in viscera and skin. In contrast, the content of As and Pb in viscera was lower, while the results of the three elements examined were similar for head samples. It should be noted that for the purpose of result comparison, data from liver and intestine samples were added and considered equivalent to our viscera side stream. Similarly, the values of gills were equated to those of whole head sample. On the other hand, the concentration of As, Hg, Cd, and Pb in the same types of side streams (including tailfins) from farmed sea bass was recently published [10]. The results of As content were lower in sea bass (0.346–1.867 µg/g) for all fish samples.

However, differences in the levels of Hg, Cd, and Pb were observed depending on each side stream. Until now, the determination of toxic metals in fish has been carried out mainly in muscle tissue due to the evaluation of risk to human health. In this sense, the content of Hg, Cd, and Pb in muscle of farmed and wild gilthead sea bream was evaluated [27]. Similar results were reported for Cd (<0.001–0.003 µg/g) and Pb (0.010–0.101 µg/g), while lower values were observed for Hg (0.002–0.047 µg/g). The authors concluded that different factors such as location, species, and seasonality influence the accumulation of Hg, Cd, and Pb. Since the limits for heavy metals in fish side streams are not currently regulated, their assessment could be carried out according to those established for edible muscle of fish (13.5 µg/g for As, 0.5 µg/g for Hg, 0.05 µg/g for Cd, and 0.30 µg/g for Pb) [6,9,26]. Considering the heavy-metal concentrations obtained, all side streams of gilthead sea bream (except for viscera) could be considered as safe raw materials to be used for the food industry according to the circular economy strategy, in terms of As, Hg, Cd, and Pb content. 

The possible occurrence of mycotoxins in muscle, head, viscera, skin, and tailfin of gilthead sea bream was also investigated. After applying a nontargeted screening approach against a spectral library containing of 223 mycotoxins and related metabolites, no mycotoxins were identified in any of the sea bream side streams studied. As far as we know, there is no information in the literature on the presence of mycotoxins in sea bream discards. Regarding the edible part of farmed sea bream, different results on mycotoxins content have been reported. There was no transfer of mycotoxins from contaminated plant-based feed to sea bream tissue [8], while emerging *Fusarium* mycotoxins such as enniantin A1, B, and B1 were identified in the muscle of some farmed fishes such as sea bream [28].

## 3. Materials and Methods

### 3.1. Reagents 

ABTS (2,2′-azinobis(3-ethylbenzothiazoline 6-sulfonic acid)), Trolox^®^ (6-hydroxy-2,5,7,8-tetramethylchroman-2-carboxylic acid), DTT (dl-dithiothreitol), diatomaceous earth (Hyflo^®^ Super Cel^®^), Trizma^®^ base, fluorescein sodium salt, and formic acid (reagent grade ≥ 95%) were purchased from Sigma-Aldrich (Steinheim, Germany). AAPH (2,2′-azobis(2-amidinopropane)) (Acros Organics), Tris (ultrapure), potassium dihydrogen phosphate, potassium sulfate, sodium phosphate dibasic, sodium chloride, *ortho*-boric acid, glycine (proteomics grade), and methanol (HPLC–MS grade) were provided by VWR International Eurolab S.L. (Barcelona, Spain). Glacial acetic acid, sulfuric acid, and sodium hydroxide were supplied by Fisher Scientific (Madrid, Spain). SDS (sodium dodecyl sulfate, purissimum-CODEX) and nitric acid (65% *p*/*p*) were obtained from Panreac (Barcelona, Spain). Acetonitrile (HPLC grade), acetone, glycerol, and bromophenol blue indicator (ACS reagent) were supplied by Merck (Darmstadt, Germany). Octadecyl C18 sorbent was from Phenomenex (Madrid, Spain), while absolute ethanol was from J.T. Baker (Deventer, The Netherlands). Anhydrous magnesium sulfate (99.5% min powder) was provided by Alfa Aesar (Karlsruhe, Germany). Deionized water (resistivity >18 MΩ/cm) was obtained through a Milli-Q SP^®^ Reagent Water System (Millipore Corporation Bedford, Bedford, MA, USA).

### 3.2. Raw Material and Sample Preparation

**Figure 4:**
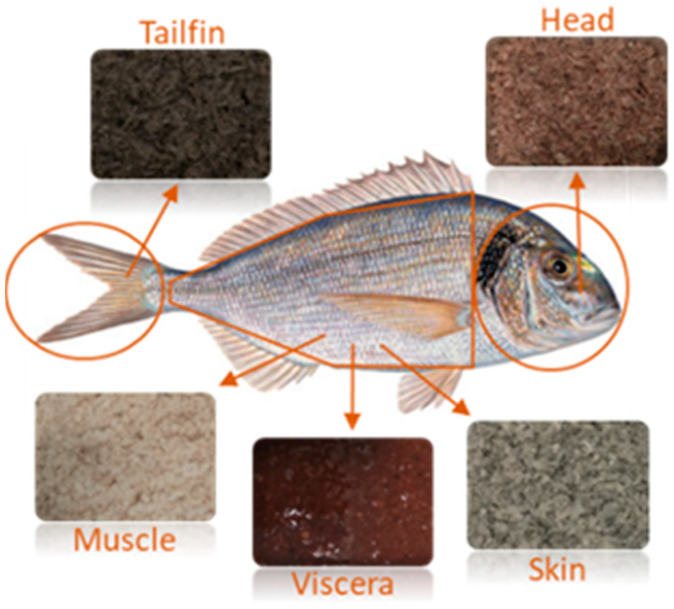
Gilthead sea bream side streams obtained to perform the analysis.

Whole gilthead sea bream fishes (*Sparus arauta*) were obtained in a local market in Valencia (Spain) during different days of April 2019. Then, they were immediately transported to the University of Valencia under refrigerated conditions. According to the commercial label, they were farmed in Greece. Individual gilthead sea bream was dissected as a simulation of fish processing for human consumption. Then, muscle remains, complete heads, viscera, flesh-free skin, and tailfins were placed separately inside aluminum containers. Next, they were frozen (−80 °C) for 48 h and freeze-dried (LABCONCO, 2.5. FREE ZONE, Kansas city, MO, USA) for 72 h. Dried samples were maintained in a desiccator until constant weight and their water content was determined. The moisture percentages were 68.77% ± 0.96%, 56.45% ± 0.91%, 32.09% ± 2.37%, 33.99% ± 0.89%, and 37.86% ± 3.85% for muscle, heads, viscera, skin, and tailfins, respectively. Similar values for gilthead sea bream muscle and head, as well as higher values for viscera and skin, were reported by Pateiro et al. [4] and Kandyliari et al. [5]. Next, the same types of side stream were ground in an analytical mill (A11 basic IKA^®^ WERKE, Staufen, Germany) and pooled together. The homogenized samples were stored at −25 °C until the extraction process and the analysis of possible contaminants. Figure 4 shows the different side streams obtained from gilthead sea bream after sample preparation.

### 3.3. Pressurized Liquid Extraction (PLE) Process

Extracts were obtained using an accelerated solvent extractor ASE 200 Dionex (Sunnyvale, CA, USA) equipped with a solvent controller. The amount of sample depended on the type of side stream. Thus, 2.5 g for muscle and head, 2 g for skin and tails, and 1.5 for viscera of gilthead sea bream were used. Then, they were mixed with diatomaceous earth and introduced into 22 mL stainless-steel cells with a glass fiber filter placed in the end part. Distilled water was used as extraction solvent, and a static extraction cycle was applied. The standard operations conditions consisted of the preheating period (1 min), heating period (5 min), flush volume (60%), and nitrogen purge (60 s). Nitrogen (145 psi) was applied to assist the pneumatic system and to purge the cells, while extractions were performed under a pressure of 1500 psi. The extraction conditions were selected according to the recently reported optimal pH–temperature–time combinations for PLE-assisted extraction to obtain antioxidant protein extracts from sea bass side streams [10]: pH 7, 20 °C, 5 min for muscle, pH 4, 60 °C, 15 min for heads, pH 7, 50 °C, 15 min for viscera, pH 7, 55 °C, 5 min for skin, and pH 7, 60 °C, 15 min for tailfins. Extracts obtained by conventional stirring using distilled water without adjusting pH or temperature and for a longer extraction time than that applied by PLE extraction were considered as controls. Then, protein recovery, protein molecular weight distribution, and total antioxidant capacity were analyzed and compared to PLE extracts. Both extraction processes (PLE and stirring) were performed in duplicate. Individual extracts were homogenized, divided into several tubes, and stored at −25 °C for subsequent analyses. 

### 3.4. Determination of Protein Recovery

The total nitrogen content in side streams of gilthead sea bream, as well as in control and PLE extracts, was determined using the Kjeldahl method [29]. Approximately 0.2 g of lyophilized fish raw material or 2 mL of fish extract was used to carry out the acid digestion prior to distillation and titration. Then, total protein content was obtained by applying the protein nitrogen conversation factor (6.25) for fish and fish side streams. In order to calculate the percentage of protein recovery, the following formula was used: (protein in extract/protein in side stream) × 100.

### 3.5. Molecular Weight Distribution of Protein Fragments

The molecular weight distribution of protein in both control (stirring) and optimal (PLE) extracts from side streams of gilthead sea bream were investigated by sodium dodecyl sulfate polyacrylamide gel electrophoresis (SDS-PAGE). Since protein concentration in fish extracts was different between samples, 100 μL for muscle extracts and 500 μL for head, viscera, skin, and tailfin extracts were used. For protein precipitation, cold acetone was added to fish extracts (4:1 *v*/*v* ratio) and they were mixed and centrifuged (11,000 rpm, 4 °C, 10 min) (Eppendorf 580 R, Thermo Fisher Scientific, Hamburg, Germany). Then, the supernatant was removed, and the pellet was dissolved in distilled water assisted by ultrasound (10 min). Next, equal volumes of SDS-PAGE sample buffer solution (62.5 mM Tris-HCl (pH 6.8), 2% SDS, 20% glycerol, 0.01% bromophenol blue, and 50 mM dithiothreitol) and protein solution were mixed and heated at 95 °C for 5 min. Afterward, 10 μL of mixture was loaded on 8–16% Mini-PROTEAN^®^ TGX™ Precast gels (Bio-Rad) and subjected to electrophoresis using a Mini-PROTEAN^®^ tetra cell (Bio-Rad). The running buffer consisted of Trizma^®^ base (25 mM), glycine (192 mM), and SDS (0.1%). The distribution of protein fragments was performed at a constant voltage (80 V) for 120 min. After electrophoresis, gels were stained in 0.125% Coomassie brilliant blue R-250 and destained in 20% methanol and 10% acetic acid until the background was as clear as possible. A standard molecular weight of protein bands from 5 to 250 kDa (Precision Plus Protein™, Bio-Rad) was used to estimate the molecular weight of protein bands. The images of the electrophoretic gels were studied using the ImageJ^®^ software, a public domain digital image processing program developed at the National Institutes of Health (NIH). Background subtraction and 8 bit format were selected for image analysis in order to improve band intensities and identify differences across protein fragments between control and PLE extracts.

### 3.6. Evaluation of Total Antioxidant Capacity

#### 3.6.1. Trolox Equivalent Antioxidant Capacity Assay (TEAC)

The TEAC assay is based on the capacity to reduce the radical cation ABTS^+^ by antioxidant compounds compared to a reference antioxidant standard (Trolox). The spectrophotometric method described by de la Fuente et al. [10] was applied. The ABTS^+^ stock solution was generated by chemical reaction with ABTS (7 mM) and K_2_S_2_O_8_ (140 mM) overnight in darkness at room temperature. Then, it was diluted in ethanol until an absorbance of 0.700 ± 0.020 at 734 nm and 30 °C to obtain the ABTS^+^ working solution. An adequate dilution of the extracts to obtain a percentage of absorbance inhibition of approximately 50% was required. Trolox standard solutions were prepared from 0 to 300 μM. The absorbance of 2 mL of ABTS^+^ working solution was considered the initial point of reaction (A_0_). Immediately, 100 μL of diluted extracts or Trolox standards were added, and the absorbance after 3 min was considered the final point of reaction (A_f_). All measures were carried out in a thermostatized ultraviolet–visible light (UV–Vis) Lambda 2 spectrophotometer (Perkin-Elmer, Jügesheim, Germany). The percentages of absorbance inhibition were calculated from the following equation: 1 − (A_f_/A_0_) × 100 and were compared to the Trolox standard curve. The results were expressed as μM Trolox equivalents.

#### 3.6.2. Oxygen Radical Absorbance Capacity Assay (ORAC)

The ORAC assay is based on the capacity of the antioxidant compounds to scavenge peroxyl radicals. The fluorometric method proposed by de la Fuente et al. [10] was used. Sodium fluorescein (0.015 mg/mL), AAPH radical solution (120 mg/mL), and Trolox standard solution (100 μM) were prepared with phosphate buffer (75 mM, pH 7). Properly diluted extracts were required. The final reaction consisted of 50 μL of diluted extract, Trolox standard, or phosphate buffer (blank), 50 μL of fluorescein, and 25 μL of AAPH. The reaction was performed at 37 °C in a Multilabel Plate Counter VICTOR3 1420 (PerkinElmer, Turku, Finland) with fluorescence filters for an excitation wavelength of 485 nm and an emission wavelength of 535 nm. The fluorescence was recorded every 5 min over 60 min (until the fluorescence in the assay was less than 5% of the initial value). The results were calculated considering the differences in areas under the fluorescence decay curve (AUC) between the blank and the sample over time. The results were expressed as μM Trolox equivalents.

### 3.7. Analysis of Heavy Metals in Gilthead Sea Bream Side Streams

The presence of As, Hg, Cd, and Pb in freeze-dried muscle, head, viscera, skin, and tailfin of gilthead sea bream was evaluated. Microwave oven-assisted digestion (MARS, CEM, Vertex, Spain) was used for the acid mineralization of fish samples. Approximately 0.30 g of side stream was placed in a Teflon reactor vessel, and 1 mL of H_2_O_2_ (30% *v*/*v*) and 4 mL of HNO_3_ (14 M) were added. The digestion was carried out by microwave irradiation with settings of 800 W, 180 °C, and 15 min. The digested samples were left to cool at room temperature and eliminate the nitrogenous vapor. Then, they were filtered through Whatman No. 1 filter paper and made up to volume with distilled water.

The identification and quantification of As, Hg, Cd, and Pb were conducted using an inductively coupled plasma spectrometer mass detector (ICP-MS model 7900, Agilent Technologies, CA, USA). The analytical conditions were as follows: carrier gas (1.07 L/min), Ar gas flow (15.0 L/min), reaction gas (He), RF power (1550 W), nebulizer pump speed (0.10 rps), and RF matching (1.80 V). Internal standard solutions of ^72^Ge, ^103^Rh, and ^193^Ir (ISC Science) at 20 µg/g were used in order to correct matrix-induced signal fluctuations and instrumental drift. Standard calibration curves from 0 to 1000 µg/L were used for the quantification of As, Cd, and Pb, while a standard calibration curve from 0 to 100 µg/L was used for Hg. Limits of detection (LODs) were calculated according to the following equation: LOD = 3sB/a where, 3sB is three times the standard deviation at zero concentration, and a is the slope of the calibration curve. LOD values were 0.0015 µg/L for Hg and Pb, 0.012 µg/L for As, and 0.004 µg/L for Cd. Distilled water was used as a blank, and the concentrations of heavy metals in the digested blank were subtracted from the values of fish samples. The results were expressed as µg of element/g of side stream in wet weight. In addition, the Certified Reference Material for Trace Metals DORM-3 (fish protein powder) was used to confirm the accuracy of the method. It was prepared and analyzed simultaneously to sea bream side streams. The recovery percentages were 98%, 86%, 76%, and 77% for As, Hg, Cd, and Pb, respectively. 

### 3.8. Analysis of Mycotoxins in Gilthead Sea Bream Side Streams

High-performance liquid chromatography coupled with electrospray ionization quadrupole time-of-flight mass spectrometry (LC-ESI-qTOF-MS) was used to analyze the mycotoxins content in freeze-dried side streams of gilthead sea bream. The chromatographic separations were achieved on an Agilent 1200-LC system (Agilent Technologies, Palo Alto, CA, USA) equipped with a Gemini^®^ column NX-C18 (3 µM, 150 × 2 mm inner diameter (ID)) (Phenomenex) and a vacuum degasser, binary pump, and autosampler. The mobile phases consisted of acidified (0.1% of formic acid) water (A) and acetonitrile (B). The gradient program was 50% B (0–6 min), followed by 100% B (7–12 min) and 50% B (13–20 min). The injection volume was 5 µL and the flow rate 0.2 mL/min. Mass spectrometry (MS) analysis was performed using a 6540 Agilent Ultra-High-Definition-Accurate-Mass-q-TOF-MS coupled to the HPLC, equipped with an Agilent Dual Jet Stream electrospray ionization (Dual AJS ESI) interface in positive and negative ionization modes. The analytical conditions were as follows: drying gas temperature (370 °C), nitrogen drying gas flow (12.0 L/min), nebulizer pressure (50 psi), fragmenter voltage (160 V), capillary voltage (3500 V), and scan range (*m*/*z* 50–1500). Automatic MS/MS experiments were carried out under the following collision energy values: *m*/*z* 100, 30 eV; *m*/*z* 500, 35 eV; *m*/*z* 1000, 40 eV; *m*/*z* 1500, 45 eV. Mass Hunter Workstation software was used for data acquisition and integration. Chromatographic separation and mass spectrometric identification conditions were based on the previously reported methodology for the analysis of mycotoxins in sea bass side streams [10].

The extraction of mycotoxins from freeze-dried side streams of gilthead sea bream was carried out using the Quick, Easy, Cheap, Effective, Rugged, and Safe (QuEChERS) method, which was employed for sea bass side streams [10]. Approximately 3 g of sample was mixed with 30 mL of acidified water (2% formic acid) in an orbital shaker (IKA KS 260) for 30 min. Next, 10 mL of acetonitrile were added, and an additional 30 min stirring was performed. Then, 8 g of MgSO_4_ and 2 g of NaCl were added to the mixture and vortexed for 30 s before centrifugation at 4000 rpm for 10 min. Afterward, 2 mL of supernatant were transferred into a 15 mL tube containing 0.1 g of Octadecyl C18 sorbent and 0.3 g of MgSO_4_. The mixture was shaken and centrifuged under the same previous conditions and the supernatant was filtered (13 mm/0.22 μm nylon filter). Lastly, 20 μL were injected into the LC-ESI-qTOF-MS system.

### 3.9. Statistical Analysis

Experimental data were subjected to one-way analysis of variance (ANOVA) to determine the significant differences among samples. Tukey’s HSD (honestly significant difference) multiple range test at a significance level of *p* < 0.05 was applied. The assumption of a normal distribution was tested using the Shapiro–Wilk test. Statistical analyses were performed with the software Statgraphics Centurion XVI.I.

## 4. Conclusions

PLE was successfully applied for the first time in this present study to obtain protein extracts with antioxidant activity from gilthead sea bream processing side streams in a sustainable way. The highest protein recovery percentage (78%) was found in viscera, while the highest antioxidant capacity was observed in muscle left over. The SDS-PAGE profiles showed differences in protein molecular weight distribution among samples. Both the levels of As, Hg, Cd, and Pb and the absence of mycotoxins in muscle, heads, viscera, skin, and tailfins of gilthead sea bream add to the limited data in the literature about these contaminants in farmed fish. One of the H2020 challenges is the recovery of proteins from natural underexploited resources in a sustainable way, and fish processing side streams may be considered great candidates for this purpose. Further research is required for both the application of sustainable technology and the utilization of fish side stream materials as a source of nutritional and bioactive compounds for the development of commercial food and feed products.

## Figures and Tables

**Table 1:** Concentration of heavy metals in gilthead sea bream side streams.

Sea Bream Side Streams	Heavy Metals (µg/g of Wet Weight)			
	As	Hg	Cd	Pb
Muscle	0.9381 ± 0.0110	0.0886 ± 0.0014	0.0008 ± 0.0001	0.0054 ± 0.0010
Head	0.8589 ± 0.0370	0.0593 ± 0.0001	0.0019 ± 0.0001	0.0190 ± 0.0002
Viscera	2.5865 ± 0.0233	0.0466 ± 0.0007	0.0683 ± 0.0007	0.0345 ± 0.0046
Skin	0.9694 ± 0.0966	0.0261 ± 0.0030	0.0101 ± 0.0003	0.0614 ± 0.0006
Tailfin	0.4406 ± 0.0055	0.0422 ± 0.0009	0.0079 ± 0.0004	0.0467 ± 0.0007
Legislation *	<13.5	<0.50	<0.05	<0.30

* Values referred to fish muscle tissue [6,9,26].

## Data Availability

Not applicable.
